# Contemporary Practices in the Assessment and Management of Climacturia: Results from an International Survey

**DOI:** 10.1016/j.euros.2026.05.004

**Published:** 2026-06-17

**Authors:** Francesco Chierigo, Bruno Bucca, Guglielmo Mantica, Giovanni Drocchi, François-Xavier Madec, Wesley Verla, Łukasz Białek, Jan Adamowicz, Andrea Cocci, Mikołaj Frankiewicz, Jakob Klemm, Paul Neuville, Maciej Oszczudłowski, Elaine J. Redmond, Clemens M. Rosenbaum, Marjan Waterloos, Felix Campos-Juanatey, David Ralph, Matteo Ferro, Malte W. Vetterlein, Trauma and Reconstructive Urology Working Party of the European Association of Urology Young Academic Urologists and the European Association of Urology Section of Genitourinary Reconstructive Surgeons, Behzad Abbasi, Leonidas Karapanos, Mattia Lo Re, Jordán Scherñuk, Juan Diego Tinajero, Mark Joseph Abalajon, Hussain M. Alnajjar, Mario Álvarez-Maestro, Carlo Bettocchi, Ozan Bozkurt, Fabiana Cancrini, Luisana Castillo Carvajal, Gökhan Çeker, Bülent Çetinel, Mehmet Gokhan Culha, Sabrina De Cillis, Muslim Dogan Deger, Rados Djinovic, Marco Falcone, Lucas Freton, Paolo Geretto, Jean-Pierre Graziana, Cyrille Guillot-Tantay, Francois Hervé, Charalampos Konstantinidis, Celeste Manfredi, Sachin Malde, José Medina-Polo, Lionel A. Micol, Andrey Morozov, Jan Novák, Yanira Ortega, Marie-Aimée Perrouin-Verbe, Arthur Peyrottes, Mikołaj Przydacz, Giorgio I. Russo, Koenraad van Renterghem, Stephane de Vergie, Patrizio Vicini, Sam Ward, Faysal Yafi

**Affiliations:** a Department of Urology, Department of Health Science, University of Milan, ASST Santi Paolo e Carlo, Milan, Italy; b Department of Urology, Sapienza University of Rome, Policlinico Umberto I Hospital, Rome, Italy; c Department of Surgical and Diagnostic Integrated Sciences (DISC), University of Genova, Genova, Italy; d Department of Urology, Ambroise Paré–Hartmann Private Hospital Group, 48 Ter Bd Victor Hugo, 92200 Neuilly-sur-Seine, Île-de-France, France; e Department of Urology, Ghent University Hospital, Ghent, Belgium; f Department of Urology, Centre of Postgraduate Medical Education, Warsaw, Poland; g Department of Regenerative Medicine, Collegium Medicum, Nicolaus Copernicus University, Bydgoszcz, Poland; h Department of Urology and Andrology, Careggi Hospital, University of Florence, Florence, Italy; i Department of Urology, Medical University of Gdańsk, Gdańsk, Poland; j Department of Urology, University Medical Center Hamburg-Eppendorf, Hamburg, Germany; k Department of Urology, Hôpital Lyon Sud, Hospices Civils de Lyon, Lyon, France; l Department of Urology, Cork University Hospital, Cork, Ireland; m Department of Urology, Asklepios Paulinen Hospital, Wiesbaden, Germany; n Department of Urology, AZ Maria Middelares, Ghent, Belgium; o Andrology and Reconstructive Urology Unit, Marqués de Valdecilla University Hospital, School of Medicine, Cantabria University, IDIVAL, Santander, Spain; p Department of Urology, University College London Hospitals (UCLH), London, United Kingdom

**Keywords:** Climacturia, Orgasm-associated incontinence, Prostate cancer, Prostatectomy, Radiotherapy, Adverse events, Survey

## Abstract

**Background:**

Climacturia is a distressing but underrecognized complication following prostate cancer treatment. Despite increasing awareness, its assessment and management remain heterogeneous in clinical practice.

**Objective:**

To evaluate current diagnostic and therapeutic practices for climacturia among expert urologists and to propose a preliminary, practice-based Climacturia Assessment Tool.

**Design, setting, and participants:**

A 34-item survey was distributed to members of the Trauma and Reconstructive Urology Working Party of the European Association of Urology (EAU) Young Academic Urologists (YAU) and the European Association of Urology Section of Genitourinary Reconstructive Surgeons (ESGURS). Fifty-one urologists and eight doctors in training completed the questionnaire. Descriptive statistics were used to summarize the findings.

**Outcome measurements and statistical analysis:**

Survey responses were summarized using medians (interquartile ranges [IQRs]) and frequencies.

**Results and limitations:**

Among the participants, 86% correctly defined climacturia, but only 59% routinely assessed it. Approaches to severity assessment were highly variable. Estimated prevalence ranged from 5% to 30%, depending on timing and association with stress urinary incontinence (SUI) or erectile dysfunction. Across 24 clinical scenarios, conservative strategies predominated in mild cases, male slings in moderate SUI-associated scenarios, artificial urinary sphincters in severe SUIs, and the Mini-Jupette procedure when penile prosthesis was required.

**Conclusions:**

Current assessment and management of climacturia vary substantially among participants. These findings highlight unmet needs in standardization and support further validation of structured assessment tools.

## Take Home Message

Expert management of climacturia is primarily driven by symptom severity and associated stress urinary incontinence or erectile dysfunction, with conservative strategies favored in mild cases and surgical interventions in more complex scenarios. Routine assessment remains inconsistent, highlighting a gap between awareness and clinical practice.

ADVANCING PRACTICE **What does this study add?**

This study provides the first international overview of real-world expert practices in the assessment and management of climacturia, identifies variability in diagnostic approaches, maps scenario-specific treatment preferences across 24 clinical situations, and proposes a preliminary assessment tool requiring prospective validation.

**Clinical Relevance**

Climacturia remains an underrecognized and inconsistently assessed complication after radical prostatectomy, despite its significant impact on patient quality of life and sexual recovery. As long-term oncologic survivorship improves, climacturia is becoming increasingly clinically relevant during extended follow-up, particularly after stress urinary incontinence and erectile dysfunction have otherwise resolved. The marked variability in current diagnostic and therapeutic approaches highlights the need for standardized assessment tools and greater clinician awareness to optimize counseling and management of climacturia.

**Patient Summary**

Climacturia remains common after prostate cancer treatment, but current treatment strategies are driven mainly by expert consensus rather than robust evidence. This study characterizes real-world practice and proposes a structured assessment tool to advance research and improve care.

## 1 Introduction

Advances in surgical and radiotherapy (RT) techniques for prostate cancer have not significantly reduced treatment-related complications, which remain a major health concern [1], [2]. Erectile dysfunction (ED) and urinary incontinence (UI) are the most prevalent complications following radical prostatectomy (RP), with reported incidence rates of 6–68% and 4–31%, respectively [3], [4]. These complications have been extensively studied, improving understanding of risk factors, mechanisms, and management strategies [5]. Consequently, patients are better informed, and urologists provide more detailed pretreatment counseling [6].

However, other functional side effects, such as climacturia and orgasmic disturbances (eg, painful orgasm, anorgasmia, and altered orgasmic sensation), have received comparatively less attention [5], [7].

Climacturia, defined by the International Society for Sexual Medicine as orgasm-associated UI occurring during ejaculation [8], [9], is a distinct form of sexual activity-related UI that can occur with or without UI. Its primary cause is neuronal injury, which disrupts bladder neck closure during climax. This is supported by studies demonstrating postradiation neuronal apoptosis and nerve damage after surgery, impairing neural health and regenerative capacity [10], [11]. Additional predictors of climacturia include penile shortening and dysorgasmia, both linked to sympathetic hyperinnervation resulting from nerve injury [12], [13], [14], [15]. These findings emphasize the shared pathophysiological mechanism of nerve damage contributing to climacturia and its associated symptoms.

First described by Lee et al in 2006, climacturia has since been reported in 20–64% of men following RP, with variability attributed to underreporting and differences in study methodologies [11], [16], [17]. Though often overlooked during follow-up, climacturia can significantly impact patients’ and their partners’ quality of life, particularly when pretreatment counseling fails to address this potential outcome [12]. The condition’s prevalence and impact highlight the need for improved recognition, assessment, and management.

Given the absence of standardized assessment tools and the variability of reported management strategies, expert consensus on real-world practice is lacking. To fill this knowledge gap, the European Association of Urology (EAU) Young Academic Urologists (YAU) Trauma and Reconstructive Urology Working Party, in collaboration with the EAU Section of Genitourinary Reconstructive Surgery (ESGURS), conducted a survey among experts in the field. The survey aimed to establish a consensus on the assessment of climacturia, to propose a novel evaluation tool, and to guide treatment strategies based on symptom severity.

## 2 Materials and methods

A structured survey (Supplementary Material 1) on climacturia was designed and distributed by the ESGURS and the EAU YAU Reconstructive Working Party.

The survey focused on demographic information, current practices regarding the evaluation and management of climacturia, and the perceived effectiveness of various treatments for postprostatectomy symptoms, including stress urinary incontinence (SUI), ED, and climacturia.

The survey targeted members of ESGURS and the EAU YAU Functional, Andrology, and Reconstructive working groups, as well as other experts in reconstructive and functional urology (hereafter referred to as “participants”). Invitations were distributed via e-mail and QR codes during the ESGURS session of the 2024 EAU Congress in Paris, France. No formal thresholds for surgical volume or years of experience were applied. Because the survey was distributed through professional networks without tracking the total number of recipients, the overall response rate could not be calculated.

The questionnaire was hosted on Google Forms, and responses were collected and exported to Microsoft Excel (Microsoft, Redmond, Washington, USA) for analysis.

Statistical analyses were predominantly descriptive. Univariable tabulations of rates, medians, and interquartile ranges (IQRs) were applied.

Based on survey responses and previous literature, we designed a Climacturia Assessment Tool (Supplementary Material 2), a simple, patient-reported scoring system designed to quantify the severity of climacturia. The tool evaluates three key dimensions—urine leakage volume (none vs small drops vs <30 ml vs ≥30 ml) and frequency of urine leakage during climax (none vs 25% of the time vs 50% of the time vs 75% of the time or always), according to what previously suggested by Mehta et al [18], and adding patient-perceived bother (not bothersome at all vs slightly bothersome vs moderately bothersome vs extremely bothersome)—each assigned a weighted score. The sum of these components generates a total score that categorizes climacturia as none, mild, moderate, or severe, allowing for standardized assessment and comparison across patients and studies. Item selection and relative weighting were empirically informed by domains consistently identified in the literature and reinforced by survey responses, rather than derived from formal statistical weighting procedures. Participants rated each component of the proposed assessment tool using a numerical Likert scale (1–9).

## 3 Results

We recorded 59 replies to the survey. Of these, 86% were fully trained urologists and 14% were doctors in training. Fifty-four (92%) participants were employed in teaching hospitals. The median age was 38 yr (IQR: 35–44.5 yr). Two-thirds of the participants (66%) reported not performing surgery specifically for climacturia. Assessment of postprostatectomy continence and erectile function was routinely performed by 95% and 100% of participants, respectively, whereas climacturia was routinely assessed by 59%. UI was most commonly quantified using either the 24-h pad test (42%) or pads per day (37%). ED was primarily evaluated using the International Index of Erectile Function (IIEF) or IIEF-5 (54%) (Table 1).

**Table 1:** Baseline characteristics of participants and summary of survey responses

Characteristic	*n* (%)/Median (IQR)
Age, yr	38 (35–44.5)
Professional status	
Staff urologists	51 (86)
Doctors in training	8 (14)
Working setting	
Teaching hospital	54 (92)
Non-teaching hospital	5 (8)
Performs surgery for male urinary incontinence (Yes)	52 (88)
Performs surgery for climacturia (Yes)	20 (34)
Dedicated clinic for patients after prostatectomy	34 (58)
Routinely asks about urinary incontinence (UI) postprostatectomy	56 (95)
Method of assessing UI	
24-h pad test	25 (42)
Pads per day	22 (37)
ICIQ-UI SF	8 (14)
ICIQ-UI SF + pads per day	2 (3)
EPIC Urinary Part	1 (2)
24-h pad test + pads per day	1 (2)
Routinely asks for erectile dysfunction postprostatectomy	59 (100)
Methods to grade erectile function	
IIEF/IIEF-5	32 (54)
Ability to achieve an erection sufficient for penetration	18 (31)
Erection hardness score (EHS)	6 (10)
IIEF-5 + EHS	1 (2)
Color Doppler penile ultrasound	1 (2)
IIEF-5 + EHS + erection sufficient for penetration	1 (2)
Routinely asks for orgasmic dysfunction	35 (60)
Definition of climacturia	
Correct	51 (86)
Partially correct	6 (10)
Incorrect	2 (4)
Thinks climacturia can present independently of stress urinary incontinence (SUI)	49 (83)
Method of assessing climacturia severity	
Combinations	13 (22.0)
– Bother + urine amount	5 (8.5)
– Bother + frequency	4 (6.8)
– Urine amount + frequency	2 (3.4)
– Holistic (patient bother + partner bother + urine amount + frequency)	2 (3.4)
Patient-reported bother alone	10 (17)
Amount of urine loss alone	10 (17)
Impact on sexual activity alone	8 (14)
Frequency alone	4 (6.8)
Impact on quality of life alone	4 (6.8)
Partner bother alone	2 (3.4)
No severity assessment	8 (14)
Grading of Climacturia Assessment Tool (Likert scale 1–9)	
Based on urine leakage volume (dry, few drops, <30 ml, and ≥30 ml)	6.0 (5.0–8.0)
Based on frequency of occurrence (never, 25%, 50%, 75%, and always)	8.0 (7.0–9.0)
Overall grading	7.0 (6.5–8.0)
Prevalence estimates 3 mo after RP	
Climacturia alone	10 (5–27.5)
Climacturia + SUI	25 (10–40)
Climacturia + erectile dysfunction (ED)	30 (20–60)
Climacturia + SUI + ED	15 (5–30)
Prevalence estimates 12 mo after RP	
Climacturia alone	5 (1–10)
Climacturia + SUI	10 (5–15)
Climacturia + ED	15 (5–30)
Climacturia + SUI + ED	10 (5–20)
Timing of climacturia surgery after RP	
12 mo or later	32 (54)
6–12 mo	9 (15)
Earlier than 6 mo	3 (5)
After failure of conservative treatment	6 (10)
Not specified	9 (15)

EPIC = Expanded Prostate Cancer Index Composite; ICIQ-UI SF = International Consultation on Incontinence Questionnaire-Urinary Incontinence Short Form; IIEF = International Index of Erectile Function; IQR = interquartile range; RP = radical prostatectomy.

Overall, 86.4% participants provided a correct definition of climacturia as orgasm-associated UI occurring during ejaculation; 10% provided a partially correct definition; and 3.4% provided an incorrect one. Furthermore, 83% stated that climacturia may occur independently of SUI.

Approaches to severity assessment were heterogeneous. A combined multi-item assessment was used by 22% of participants, while others relied mainly on patient bother (17%), urine leakage volume (17%), impact on sexual activity (14%), frequency (6.8%), partner bother (3.4%), or quality-of-life impairment (3.4%). In 14% of cases, no structured method was reported. Responders graded the components of urine volume and frequency with median scores of 6.0 (IQR: 5–8) and 8.0 (IQR: 7–9), respectively, while the overall grading of the tool was 7.0 (IQR: 6.5–8.0) (Table 1).

At 3 mo postprostatectomy, participants estimated a median prevalence of 10% (IQR: 5–27.5) for isolated climacturia, 25% (IQR: 10–40) for climacturia associated with SUI, 30% (IQR: 20–60) for climacturia associated with ED, and 15% (IQR: 5–30) for climacturia associated with both SUI and ED. At 12 mo, the estimated prevalence was 5% (IQR: 3–15) for isolated climacturia and remained between 10% and 15% for all combined forms (Table 1).

Regarding timing for intervention, 54% of participants considered surgical management only after at least 12 mo from prostatectomy, 15% between 6 and 12 mo, and 5.1% within the first 6 mo. Another 10% reported intervening after failure of conservative therapy without specifying an interval (Table 1).

**Fig. 1:**
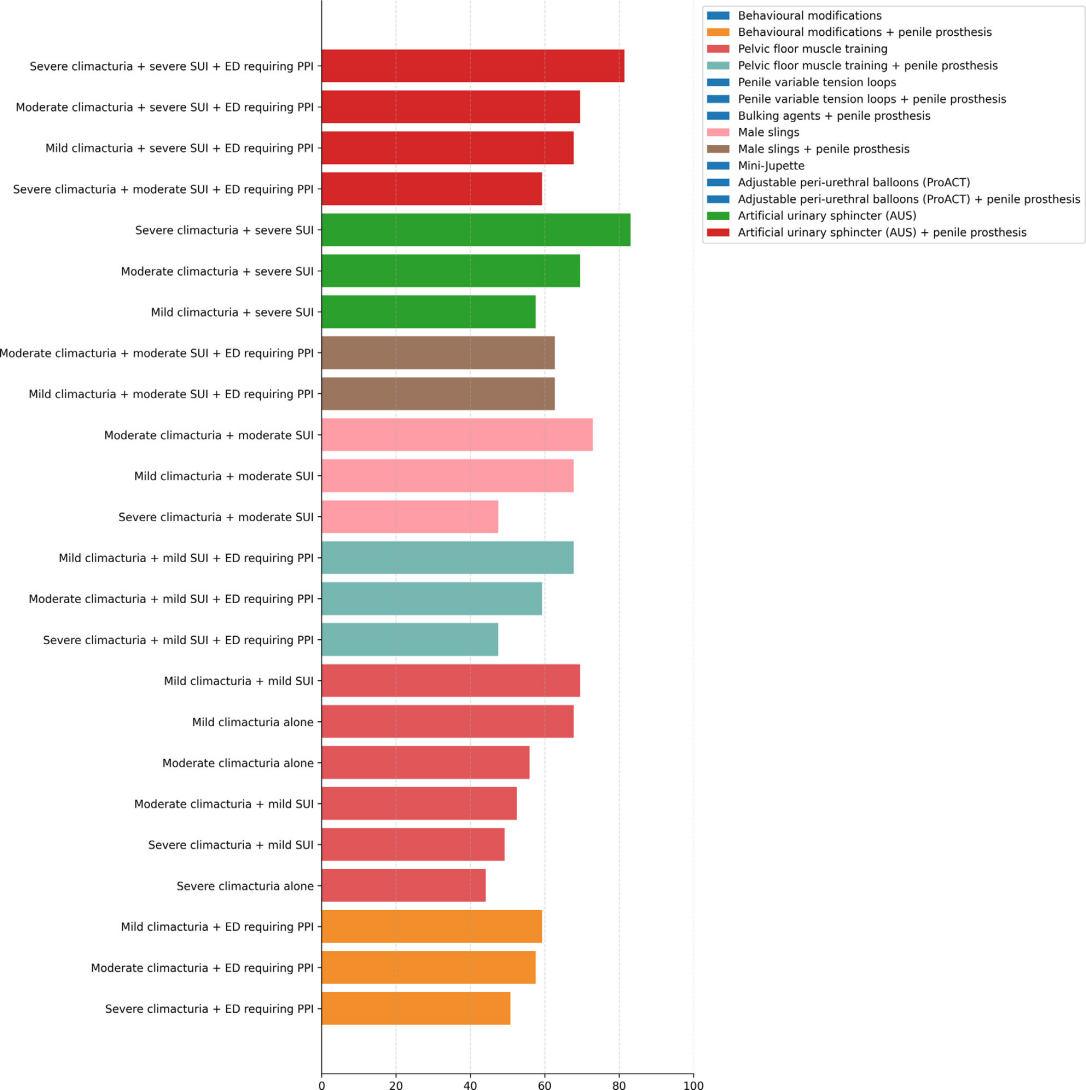
Most commonly selected management strategy for each combination of different severity levels (mild, moderate, and severe) of climacturia, stress urinary incontinence (SUI), and erectile dysfunction (ED). This figure demonstrates a stepwise escalation in expert recommendations, with conservative approaches predominating in mild presentations, male slings favored in moderate SUI-associated scenarios, artificial urinary sphincter in severe SUI, and Mini-Jupette emerging as the preferred option when penile prosthesis is required. PPI = penile prosthesis implantation.

**Fig. 2:**
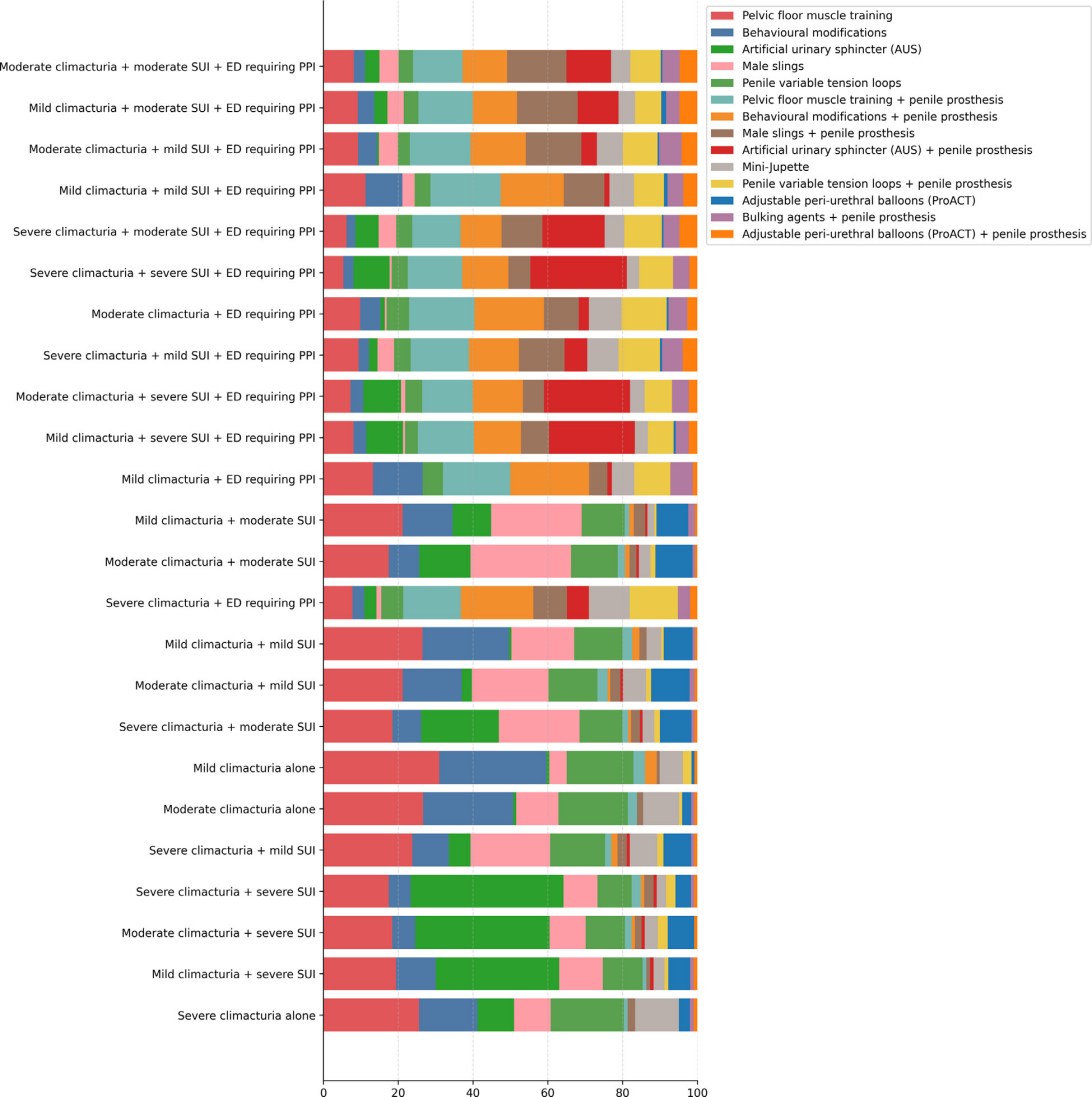
Distribution of treatment strategies across combinations of different severity levels (mild, moderate, and severe) of climacturia, stress urinary incontinence (SUI), and erectile dysfunction (ED). The distribution illustrates substantial heterogeneity in treatment selection while highlighting that therapeutic choices are primarily driven by the severity of coexisting SUI or the need for penile prosthesis rather than climacturia severity alone. PPI = penile prosthesis implantation.

Treatment selections across the 24 clinical scenarios are summarized in the matrix (Table 2, Table 3, Fig. 1, Fig. 2, Supplementary Material 3–16).

**Table 2:** Distribution of expert recommendations for the management of climacturia according to symptom severity and associated conditions

**a) For which patient would you consider behavioral modifications (condoms, emptying the bladder before intercourse, etc.)?**	
Category	*N* (%)
Mild climacturia alone	37 (63)
Moderate climacturia alone	30 (51)
Severe climacturia alone	16 (27)
Mild climacturia + mild SUI	36 (61)
Mild climacturia + moderate SUI	22 (37)
Mild climacturia + severe SUI	11 (19)
Moderate climacturia + mild SUI	23 (39)
Moderate climacturia + moderate SUI	13 (22)
Moderate climacturia + severe SUI	7 (12)
Severe climacturia + mild SUI	12 (20)
Severe climacturia + moderate SUI	10 (17)
Severe climacturia + severe SUI	7 (12)
Mild climacturia + ED requiring PPI	22 (37)
Moderate climacturia + ED requiring PPI	10 (17)
Severe climacturia + ED requiring PPI	5 (8.5)
Mild climacturia + mild SUI + ED requiring PPI	21 (36)
Mild climacturia + moderate SUI + ED requiring PPI	10 (17)
Mild climacturia + severe SUI + ED requiring PPI	6 (10)
Moderate climacturia + mild SUI + ED requiring PPI	11 (19)
Moderate climacturia + moderate SUI + ED requiring PPI	7 (12)
Moderate climacturia + severe SUI + ED requiring PPI	6 (10)
Severe climacturia + mild SUI + ED requiring PPI	5 (8.5)
Severe climacturia + moderate SUI + ED requiring PPI	5 (8.5)
Severe climacturia + severe SUI + ED requiring PPI	5 (8.5)

**b) For which patient would you consider behavioral modifications (condoms, emptying the bladder before intercourse, etc.) + penile prosthesis implantation?**	
Category	*N* (%)
Mild climacturia alone	4 (6.8)
Moderate climacturia alone	0 (0.0)
Severe climacturia alone	0 (0.0)
Mild climacturia + mild SUI	3 (5.1)
Mild climacturia + moderate SUI	2 (3.4)
Mild climacturia + severe SUI	0 (0.0)
Moderate climacturia + mild SUI	1 (1.7)
Moderate climacturia + moderate SUI	2 (3.4)
Moderate climacturia + severe SUI	1 (1.7)
Severe climacturia + mild SUI	2 (3.4)
Severe climacturia + moderate SUI	1 (1.7)
Severe climacturia + severe SUI	1 (1.7)
Mild climacturia + ED requiring PPI	35 (59)
Moderate climacturia + ED requiring PPI	34 (58)
Severe climacturia + ED requiring PPI	30 (51)
Mild climacturia + mild SUI + ED requiring PPI	36 (61)
Mild climacturia + moderate SUI + ED requiring PPI	27 (46)
Mild climacturia + severe SUI + ED requiring PPI	22 (37)
Moderate climacturia + mild SUI + ED requiring PPI	32 (54)
Moderate climacturia + moderate SUI + ED requiring PPI	28 (48)
Moderate climacturia + severe SUI + ED requiring PPI	24 (41)
Severe climacturia + mild SUI + ED requiring PPI	24 (41)
Severe climacturia + moderate SUI + ED requiring PPI	23 (39)
Severe climacturia + severe SUI + ED requiring PPI	23 (39)

**c) For which patient would you consider pelvic floor muscle training?**	
Category	*N* (%)
Mild climacturia alone	40 (68)
Moderate climacturia alone	33 (56)
Severe climacturia alone	26 (44)
Mild climacturia + mild SUI	41 (70)
Mild climacturia + moderate SUI	35 (59)
Mild climacturia + severe SUI	20 (34)
Moderate climacturia + mild SUI	31 (53)
Moderate climacturia + moderate SUI	28 (48)
Moderate climacturia + severe SUI	21 (36)
Severe climacturia + mild SUI	29 (49)
Severe climacturia + moderate SUI	24 (41)
Severe climacturia + severe SUI	21 (36)
Mild climacturia + ED requiring PPI	22 (37)
Moderate climacturia + ED requiring PPI	18 (31)
Severe climacturia + ED requiring PPI	12 (21)
Mild climacturia + mild SUI + ED requiring PPI	24 (41)
Mild climacturia + moderate SUI + ED requiring PPI	21 (36)
Mild climacturia + severe SUI + ED requiring PPI	14 (24)
Moderate climacturia + mild SUI + ED requiring PPI	20 (34)
Moderate climacturia + moderate SUI + ED requiring PPI	19 (32)
Moderate climacturia + severe SUI + ED requiring PPI	13 (22)
Severe climacturia + mild SUI + ED requiring PPI	17 (29)
Severe climacturia + moderate SUI + ED requiring PPI	13 (22)
Severe climacturia + severe SUI + ED requiring PPI	10 (17)

**d) For which patient would you consider pelvic floor muscle training + penile prosthesis implantation?**	
Category	*N* (%)
Mild climacturia alone	4 (6.8)
Moderate climacturia alone	3 (5.1)
Severe climacturia alone	1 (1.7)
Mild climacturia + mild SUI	4 (6.8)
Mild climacturia + moderate SUI	2 (3.4)
Mild climacturia + severe SUI	1 (1.7)
Moderate climacturia + mild SUI	4 (6.8)
Moderate climacturia + moderate SUI	3 (5.1)
Moderate climacturia + severe SUI	2 (3.4)
Severe climacturia + mild SUI	2 (3.4)
Severe climacturia + moderate SUI	2 (3.4)
Severe climacturia + severe SUI	3 (5.1)
Mild climacturia + ED requiring PPI	30 (51)
Moderate climacturia + ED requiring PPI	32 (54)
Severe climacturia + ED requiring PPI	24 (41)
Mild climacturia + mild SUI + ED requiring PPI	40 (68)
Mild climacturia + moderate SUI + ED requiring PPI	33 (56)
Mild climacturia + severe SUI + ED requiring PPI	26 (44)
Moderate climacturia + mild SUI + ED requiring PPI	35 (59)
Moderate climacturia + moderate SUI + ED requiring PPI	31 (53)
Moderate climacturia + severe SUI + ED requiring PPI	24 (41)
Severe climacturia + mild SUI + ED requiring PPI	28 (48)
Severe climacturia + moderate SUI + ED requiring PPI	27 (46)
Severe climacturia + severe SUI + ED requiring PPI	27 (46)

**e) For which patient would you consider penile variable tension loops/soft silicone tension loops?**	
Category	*N* (%)
Mild climacturia alone	23 (39)
Moderate climacturia alone	23 (39)
Severe climacturia alone	20 (34)
Mild climacturia + mild SUI	20 (34)
Mild climacturia + moderate SUI	19 (32)
Mild climacturia + severe SUI	11 (19)
Moderate climacturia + mild SUI	19 (32)
Moderate climacturia + moderate SUI	20 (34)
Moderate climacturia + severe SUI	12 (20)
Severe climacturia + mild SUI	18 (31)
Severe climacturia + moderate SUI	15 (25)
Severe climacturia + severe SUI	11 (19)
Mild climacturia + ED requiring PPI	9 (15)
Moderate climacturia + ED requiring PPI	11 (19)
Severe climacturia + ED requiring PPI	9 (15)
Mild climacturia + mild SUI + ED requiring PPI	9 (15)
Mild climacturia + moderate SUI + ED requiring PPI	9 (15)
Mild climacturia + severe SUI + ED requiring PPI	6 (10)
Moderate climacturia + mild SUI + ED requiring PPI	7 (12)
Moderate climacturia + moderate SUI + ED requiring PPI	9 (15)
Moderate climacturia + severe SUI + ED requiring PPI	8 (14)
Severe climacturia + mild SUI + ED requiring PPI	8 (14)
Severe climacturia + moderate SUI + ED requiring PPI	9 (15)
Severe climacturia + severe SUI + ED requiring PPI	8 (14)

**f) For which patient would you consider penile variable tension loops/soft silicone tension loops + penile prosthesis implantation?**	
Category	*N* (%)
Mild climacturia alone	3 (5.1)
Moderate climacturia alone	1 (1.7)
Severe climacturia alone	0 (0)
Mild climacturia + mild SUI	1 (1.7)
Mild climacturia + moderate SUI	1 (1.7)
Mild climacturia + severe SUI	1 (1.7)
Moderate climacturia + mild SUI	2 (3.4)
Moderate climacturia + moderate SUI	2 (3.4)
Moderate climacturia + severe SUI	3 (5.1)
Severe climacturia + mild SUI	2 (3.4)
Severe climacturia + moderate SUI	2 (3.4)
Severe climacturia + severe SUI	3 (5.1)
Mild climacturia + ED requiring PPI	16 (27)
Moderate climacturia + ED requiring PPI	22 (37)
Severe climacturia + ED requiring PPI	20 (34)
Mild climacturia + mild SUI + ED requiring PPI	17 (29)
Mild climacturia + moderate SUI + ED requiring PPI	16 (27)
Mild climacturia + severe SUI + ED requiring PPI	12 (20)
Moderate climacturia + mild SUI + ED requiring PPI	20 (34)
Moderate climacturia + moderate SUI + ED requiring PPI	19 (32)
Moderate climacturia + severe SUI + ED requiring PPI	13 (22)
Severe climacturia + mild SUI + ED requiring PPI	20 (34)
Severe climacturia + moderate SUI + ED requiring PPI	21 (36)
Severe climacturia + severe SUI + ED requiring PPI	17 (29)

**g) For which patient would you consider bulking agents + penile prosthesis implantation?**	
Category	*N* (%)
Mild climacturia alone	0 (0)
Moderate climacturia alone	1 (1.7)
Severe climacturia alone	1 (1.7)
Mild climacturia + mild SUI	1 (1.7)
Mild climacturia + moderate SUI	3 (5.1)
Mild climacturia + severe SUI	1 (1.7)
Moderate climacturia + mild SUI	2 (3.4)
Moderate climacturia + moderate SUI	1 (1.7)
Moderate climacturia + severe SUI	0 (0)
Severe climacturia + mild SUI	1 (1.7)
Severe climacturia + moderate SUI	1 (1.7)
Severe climacturia + severe SUI	1 (1.7)
Mild climacturia + ED requiring PPI	10 (17)
Moderate climacturia + ED requiring PPI	9 (15)
Severe climacturia + ED requiring PPI	5 (8.5)
Mild climacturia + mild SUI + ED requiring PPI	9 (15)
Mild climacturia + moderate SUI + ED requiring PPI	8 (14)
Mild climacturia + severe SUI + ED requiring PPI	6 (10)
Moderate climacturia + mild SUI + ED requiring PPI	13 (22)
Moderate climacturia + moderate SUI + ED requiring PPI	11 (19)
Moderate climacturia + severe SUI + ED requiring PPI	8 (14)
Severe climacturia + mild SUI + ED requiring PPI	10 (17)
Severe climacturia + moderate SUI + ED requiring PPI	9 (15)
Severe climacturia + severe SUI + ED requiring PPI	8 (14)

**h) For which patient would you consider male slings?**	
Category	*N* (%)
Mild climacturia alone	6 (10)
Moderate climacturia alone	14 (24)
Severe climacturia alone	10 (17)
Mild climacturia + mild SUI	26 (44)
Mild climacturia + moderate SUI	40 (68)
Mild climacturia + severe SUI	12 (20)
Moderate climacturia + mild SUI	30 (51)
Moderate climacturia + moderate SUI	43 (73)
Moderate climacturia + severe SUI	11 (19)
Severe climacturia + mild SUI	26 (44)
Severe climacturia + moderate SUI	28 (48)
Severe climacturia + severe SUI	11 (19)
Mild climacturia + ED requiring PPI	0 (0)
Moderate climacturia + ED requiring PPI	1 (1.7)
Severe climacturia + ED requiring PPI	2 (3.4)
Mild climacturia + mild SUI + ED requiring PPI	7 (12)
Mild climacturia + moderate SUI + ED requiring PPI	10 (17)
Mild climacturia + severe SUI + ED requiring PPI	1 (1.7)
Moderate climacturia + mild SUI + ED requiring PPI	11 (19)
Moderate climacturia + moderate SUI + ED requiring PPI	12 (20)
Moderate climacturia + severe SUI + ED requiring PPI	2 (3.4)
Severe climacturia + mild SUI + ED requiring PPI	8 (14)
Severe climacturia + moderate SUI + ED requiring PPI	10 (17)
Severe climacturia + severe SUI + ED requiring PPI	1 (1.7)

**i) For which patient would you consider male slings + penile prosthesis implantation?**	
Category	*N* (%)
Mild climacturia alone	1 (1.7)
Moderate climacturia alone	2 (3.)
Severe climacturia alone	2 (3.4)
Mild climacturia + mild SUI	3 (5.1)
Mild climacturia + moderate SUI	5 (8.5)
Mild climacturia + severe SUI	1 (1.7)
Moderate climacturia + mild SUI	4 (6.8)
Moderate climacturia + moderate SUI	3 (5.1)
Moderate climacturia + severe SUI	2 (3.4)
Severe climacturia + mild SUI	3 (5.1)
Severe climacturia + moderate SUI	3 (5.1)
Severe climacturia + severe SUI	3 (5.1)
Mild climacturia + ED requiring PPI	8 (14)
Moderate climacturia + ED requiring PPI	17 (29)
Severe climacturia + ED requiring PPI	14 (24)
Mild climacturia + mild SUI + ED requiring PPI	23 (39)
Mild climacturia + moderate SUI + ED requiring PPI	37 (63)
Mild climacturia + severe SUI + ED requiring PPI	13 (22)
Moderate climacturia + mild SUI + ED requiring PPI	32 (54)
Moderate climacturia + moderate SUI + ED requiring PPI	37 (63)
Moderate climacturia + severe SUI + ED requiring PPI	10 (19)
Severe climacturia + mild SUI + ED requiring PPI	22 (37)
Severe climacturia + moderate SUI + ED requiring PPI	23 (39)
Severe climacturia + severe SUI + ED requiring PPI	11 (19)

**j) For which patient would you consider Mini-Jupette?**	
Category	*N* (%)
Mild climacturia alone	8 (14)
Moderate climacturia alone	12 (20)
Severe climacturia alone	12 (20)
Mild climacturia + mild SUI	6 (10)
Mild climacturia + moderate SUI	3 (5.1)
Mild climacturia + severe SUI	3 (5.1)
Moderate climacturia + mild SUI	9 (15)
Moderate climacturia + moderate SUI	5 (8.5)
Moderate climacturia + severe SUI	4 (6.8)
Severe climacturia + mild SUI	9 (15)
Severe climacturia + moderate SUI	4 (6.8)
Severe climacturia + severe SUI	3 (5.1)
Mild climacturia + ED requiring PPI	10 (17)
Moderate climacturia + ED requiring PPI	16 (27)
Severe climacturia + ED requiring PPI	17 (29)
Mild climacturia + mild SUI + ED requiring PPI	14 (24)
Mild climacturia + moderate SUI + ED requiring PPI	10 (17)
Mild climacturia + severe SUI + ED requiring PPI	6 (10)
Moderate climacturia + mild SUI + ED requiring PPI	15 (25)
Moderate climacturia + moderate SUI + ED requiring PPI	12 (20)
Moderate climacturia + severe SUI + ED requiring PPI	7 (12)
Severe climacturia + mild SUI + ED requiring PPI	15 (25)
Severe climacturia + moderate SUI + ED requiring PPI	11 (19)
Severe climacturia + severe SUI + ED requiring PPI	6 (10)

**k) For which patient would you consider adjustable peri-urethral balloons (ie, ProACT)?**	
Category	*N* (%)
Mild climacturia alone	1 (1.7)
Moderate climacturia alone	3 (5.1)
Severe climacturia alone	3 (5.1)
Mild climacturia + mild SUI	12 (20)
Mild climacturia + moderate SUI	14 (24)
Mild climacturia + severe SUI	6 (10)
Moderate climacturia + mild SUI	15 (25)
Moderate climacturia + moderate SUI	16 (27)
Moderate climacturia + severe SUI	8 (1)
Severe climacturia + mild SUI	9 (15)
Severe climacturia + moderate SUI	11 (19)
Severe climacturia + severe SUI	5 (8.5)
Mild climacturia + ED requiring PPI	0 (0)
Moderate climacturia + ED requiring PPI	1 (1.7)
Severe climacturia + ED requiring PPI	0 (0)
Mild climacturia + mild SUI + ED requiring PPI	2 (3.4)
Mild climacturia + moderate SUI + ED requiring PPI	3 (5.1)
Mild climacturia + severe SUI + ED requiring PPI	1 (1.7)
Moderate climacturia + mild SUI + ED requiring PPI	1 (1.7)
Moderate climacturia + moderate SUI + ED requiring PPI	1 (1.7)
Moderate climacturia + severe SUI + ED requiring PPI	0 (0)
Severe climacturia + mild SUI + ED requiring PPI	1 (1.7)
Severe climacturia + moderate SUI + ED requiring PPI	1 (1.7)
Severe climacturia + severe SUI + ED requiring PPI	0 (0)

**l) For which patient would you consider adjustable peri-urethral balloons (ie, ProACT) + penile prosthesis implantation?**	
Category	*N* (%)
Mild climacturia alone	1 (1.7)
Moderate climacturia alone	1 (1.7)
Severe climacturia alone	1 (1.7)
Mild climacturia + mild SUI	1 (1.7)
Mild climacturia + moderate SUI	1 (1.7)
Mild climacturia + severe SUI	1 (1.7)
Moderate climacturia + mild SUI	1 (1.7)
Moderate climacturia + moderate SUI	1 (1.7)
Moderate climacturia + severe SUI	1 (1.7)
Severe climacturia + mild SUI	1 (1.7)
Severe climacturia + moderate SUI	1 (1.7)
Severe climacturia + severe SUI	1 (1.7)
Mild climacturia + ED requiring PPI	2 (3.4)
Moderate climacturia + ED requiring PPI	5 (8.5)
Severe climacturia + ED requiring PPI	3 (5.1)
Mild climacturia + mild SUI + ED requiring PPI	8 (14)
Mild climacturia + moderate SUI + ED requiring PPI	11 (19)
Mild climacturia + severe SUI + ED requiring PPI	4 (6.8)
Moderate climacturia + mild SUI + ED requiring PPI	9 (15)
Moderate climacturia + moderate SUI + ED requiring PPI	11 (19)
Moderate climacturia + severe SUI + ED requiring PPI	4 (6.8)
Severe climacturia + mild SUI + ED requiring PPI	7 (12)
Severe climacturia + moderate SUI + ED requiring PPI	10 (19)
Severe climacturia + severe SUI + ED requiring PPI	4 (6.8)

**m) For which patient would you consider artificial urinary sphincter (AUS)?**	
Category	*N* (%)
Mild climacturia alone	1 (1.7)
Moderate climacturia alone	1 (1.7)
Severe climacturia alone	10 (17)
Mild climacturia + mild SUI	1 (1.7)
Mild climacturia + moderate SUI	17 (29)
Mild climacturia + severe SUI	34 (58)
Moderate climacturia + mild SUI	4 (6.8)
Moderate climacturia + moderate SUI	22 (37)
Moderate climacturia + severe SUI	41 (70)
Severe climacturia + mild SUI	7 (12)
Severe climacturia + moderate SUI	27 (46)
Severe climacturia + severe SUI	49 (83)
Mild climacturia + ED requiring PPI	0 (0)
Moderate climacturia + ED requiring PPI	2 (3.4)
Severe climacturia + ED requiring PPI	5 (8.5)
Mild climacturia + mild SUI + ED requiring PPI	0 (0)
Mild climacturia + moderate SUI + ED requiring PPI	8 (14)
Mild climacturia + severe SUI + ED requiring PPI	17 (29)
Moderate climacturia + mild SUI + ED requiring PPI	1 (1.7)
Moderate climacturia + moderate SUI + ED requiring PPI	9 (15)
Moderate climacturia + severe SUI + ED requiring PPI	18 (31)
Severe climacturia + mild SUI + ED requiring PPI	4 (6.8)
Severe climacturia + moderate SUI + ED requiring PPI	13 (22)
Severe climacturia + severe SUI + ED requiring PPI	18 (31)

**n) For which patient would you consider artificial urinary sphincter (AUS) + penile prosthesis implantation?**	
Category	*N* (%)
Mild climacturia alone	0 (0)
Moderate climacturia alone	0 (0)
Severe climacturia alone	0 (0)
Mild climacturia + mild SUI	0 (0)
Mild climacturia + moderate SUI	1 (1.7)
Mild climacturia + severe SUI	1 (1.7)
Moderate climacturia + mild SUI	1 (1.7)
Moderate climacturia + moderate SUI	1 (1.7)
Moderate climacturia + severe SUI	1 (1.7)
Severe climacturia + mild SUI	1 (1.7)
Severe climacturia + moderate SUI	1 (1.7)
Severe climacturia + severe SUI	1 (1.7)
Mild climacturia + ED requiring PPI	2 (3.4)
Moderate climacturia + ED requiring PPI	5 (8.5)
Severe climacturia + ED requiring PPI	9 (15)
Mild climacturia + mild SUI + ED requiring PPI	3 (5.1)
Mild climacturia + moderate SUI + ED requiring PPI	25 (42)
Mild climacturia + severe SUI + ED requiring PPI	40 (68)
Moderate climacturia + mild SUI + ED requiring PPI	9 (15)
Moderate climacturia + moderate SUI + ED requiring PPI	28 (48)
Moderate climacturia + severe SUI + ED requiring PPI	41 (70)
Severe climacturia + mild SUI + ED requiring PPI	11 (19)
Severe climacturia + moderate SUI + ED requiring PPI	35 (60)
Severe climacturia + severe SUI + ED requiring PPI	48 (81)

ED = erectile dysfunction; PPI = penile prosthesis implantation; SUI = stress urinary incontinence.

**Table 3:** Unified matrix summarizing expert treatment preferences for climacturia across different clinical scenarios (*N* [%])

	BM	BM + PPI	PFMT	PFMT + PPI	PVTL	PVTL + PPI	BA + PPI	MS	MS + PPI	MJ	ProACT	ProACT + PPI	AUS	AUS + PPI
Mild climacturia alone (*N* [%])	37 (63)	4 (6.8)	40 (68)	4 (6.8)	23 (39)	3 (5.1)	0 (0)	6 (10)	1 (1.7)	8 (14)	1 (1.7)	1 (1.7)	1 (1.7)	0 (0.0)
Moderate climacturia alone (*N* [%])	30 (51)	0 (0.0)	33 (56)	3 (5.1)	23 (39)	1 (1.7)	1 (1.7)	14 (24)	2 (3.4)	12 (20)	3 (5.1)	1 (1.7)	1 (1.7)	0 (0.0)
Severe climacturia alone (*N* [%])	16 (27)	0 (0)	26 (44)	1 (1.7)	20 (34)	0 (0)	1 (1.7)	10 (17)	2 (3.4)	12 (20)	3 (5.1)	1 (1.7)	10 (17)	0 (0.0)
Mild climacturia + mild SUI (*N* [%])	36 (61)	3 (5.1)	41 (70)	4 (6.8)	20 (34)	1 (1.7)	1 (1.7)	26 (44)	3 (5.1)	6 (10)	12 (20)	1 (1.7)	1 (1.7)	0 (0.0)
Mild climacturia + moderate SUI (*N* [%])	22 (37)	2 (3.4)	35 (59)	2 (3.4)	19 (32)	1 (1.7)	3 (5.1)	40 (68)	5 (8.5)	3 (5.1)	14 (24)	1 (1.7)	17 (29)	1 (1.7)
Mild climacturia + severe SUI (*N* [%])	11 (19)	0 (0)	20 (34)	1 (1.7)	11 (19)	1 (1.7)	1 (1.7)	12 (20)	1 (1.7)	3 (5.1)	6 (10)	1 (1.7)	34 (58)	1 (1.7)
Moderate climacturia + mild SUI (*N* [%])	23 (39)	1 (1.7)	31 (53)	4 (6.8)	19 (3)	2 (3.4)	2 (3.4)	30 (51)	4 (6.8)	9 (1)	15 (25)	1 (1.)	4 (6.8)	1 (1.7)
Moderate climacturia + moderate SUI (*N* [%])	13 (2)	2 (3.4)	28 (48)	3 (5.1)	20 (34)	2 (3.4)	1 (1.7)	43 (73)	3 (5.)	5 (8.5)	16 (2)	1 (1.7)	22 (37)	1 (1.7)
Moderate climacturia + severe SUI (*N* [%])	7 (12)	1 (1.7)	21 (36)	2 (3.4)	12 (20)	3 (5.1)	0 (0)	11 (1)	2 (3.4)	4 (6.8)	8 (14)	1 (1.)	41 (70)	1 (1.7)
Severe climacturia + mild SUI (*N* [%])	12 (20)	2 (3.4)	29 (49)	2 (3.4)	18 (31)	2 (3.4)	1 (1.7)	26 (44)	3 (5.1)	9 (15)	9 (15)	1 (1.7)	7 (12)	1 (1.7)
Severe climacturia + moderate SUI (*N* [%])	10 (17)	1 (1.7)	24 (41)	2 (3.4)	15 (25)	2 (3.4)	1 (1.7)	28 (48)	3 (5.1)	4 (6.8)	11 (19)	1 (1.7)	27 (46)	1 (1.7)
Severe climacturia + severe SUI (*N* [%])	7 (12)	1 (1.7)	21 (36)	3 (5.1)	11 (19)	3 (5.1)	1 (1.7)	11 (19)	3 (5.1)	3 (5.1)	5 (8.5)	1 (1.7)	49 (83)	1 (1.7)
Mild climacturia + ED requiring PPI (*N* [%])	22 (37)	35 (59)	22 (37)	30 (51)	9 (15)	16 (27)	10 (17)	0 (0)	8 (14)	10 (17)	0 (0)	2 (3.4)	0 (0)	2 (3.4)
Moderate climacturia + ED requiring PPI (*N* [%])	10 (17)	34 (58)	18 (31)	32 (54)	11 (19)	22 (37)	9 (15)	1 (1.7)	17 (29)	16 (27)	1 (1.7)	5 (8.5)	2 (3.4)	5 (8.5)
Severe climacturia + ED requiring PPI (*N* [%])	5 (8.5)	30 (51)	12 (20)	24 (41)	9 (15)	20 (34)	5 (8.5)	2 (3.4)	14 (24)	17 (29)	0 (0)	3 (5.1)	5 (8.5)	9 (15)
Mild climacturia + mild SUI + ED requiring PPI (*N* [%])	21 (36)	36 (61)	24 (41)	40 (68)	9 (15)	17 (29)	9 (15)	7 (12)	23 (39)	14 (24)	2 (3.4)	8 (14)	0 (0)	3 (5.1)
Mild climacturia + moderate SUI + ED requiring PPI (*N* [%])	10 (17)	27 (46)	21 (36)	33 (56)	9 (15)	16 (27)	8 (14)	10 (17)	37 (63)	10 (17)	3 (5.1)	11 (19)	8 (14)	25 (42)
Mild climacturia + severe SUI + ED requiring PPI (*N* [%])	6 (10)	22 (37)	14 (24)	26 (44)	6 (10)	12 (20)	6 (10)	1 (1.7)	13 (22)	6 (10)	1 (1.7)	4 (6.8)	17 (29)	40 (68)
Moderate climacturia + mild SUI + ED requiring PPI (*N* [%])	11 (19)	32 (54)	20 (34)	35 (60)	7 (12)	20 (34)	13 (22)	11 (19)	32 (54)	15 (25)	1 (1.7)	9 (15)	1 (1.7)	9 (15)
Moderate climacturia + moderate SUI + ED requiring PPI (*N* [%])	7 (12)	28 (48)	19 (32)	31 (52)	9 (15)	19 (32)	11 (19)	12 (20)	37 (63)	12 (20)	1 (1.7)	11 (19)	9 (15)	28 (48)
Moderate climacturia + severe SUI + ED requiring PPI (*N* [%])	6 (10)	24 (41)	13 (22)	24 (41)	8 (14)	13 (22)	8 (14)	2 (3.4)	10 (17)	7 (12)	0 (0)	4 (6.8)	18 (31)	41 (70)
Severe climacturia + mild SUI + ED requiring PPI (*N* [%])	5 (8.5)	24 (41)	17 (29)	28 (48)	8 (14)	20 (34)	10 (17)	8 (14)	22 (37)	15 (25)	1 (1.7)	7 (12)	4 (6.8)	11 (19)
Severe climacturia + moderate SUI + ED requiring PPI (*N* [%])	5 (8.5)	23 (39)	13 (22)	27 (46)	9 (15)	21 (36)	9 (15)	10 (17)	23 (39)	11 (19)	1 (1.7)	10 (17)	13 (22)	35 (60)
Severe climacturia + severe SUI + ED requiring PPI (*N* [%])	5 (8.5)	23 (39)	10 (17)	27 (46)	8 (14)	17 (29)	8 (14)	1 (1.7)	11 (19)	6 (10)	0 (0)	4 (6.8)	18 (31)	48 (81)

AUS = artificial urinary sphincter; BA = bulking agents; BM = behavioral modifications; ED = erectile dysfunction; MJ = Mini-Jupette; MS = male slings; PFMT = pelvic floor muscle training; PPI = penile prosthesis implantation; ProACT = adjustable peri-urethral balloons; PVTL = penile variable tension loops, SUI = stress urinary incontinence.

In scenarios involving mild climacturia, whether isolated or associated with mild SUI or ED, the most frequently selected treatments were behavioral modifications, pelvic floor muscle training, and penile variable tension loops. For example, in “mild climacturia alone,” pelvic floor muscle training was selected by 68% (40/59) and behavioral modifications by 63% (37/59). In “mild climacturia + mild SUI,” pelvic floor muscle training was selected by 63% (37/59) and behavioral modifications by 61% (36/59), whereas penile variable tension loops were selected by 56% (33/59).

In scenarios combining moderate climacturia with mild or moderate SUI, male slings showed the highest frequencies. In “moderate climacturia + mild SUI,” T8 was selected by 73% (43/59), while in “moderate climacturia + moderate SUI,” T8 was selected by 70% (41/59).

Across all scenarios involving severe SUI, the artificial urinary sphincter (AUS), either alone or in combination with penile prosthesis implantation (PPI), represented the most frequently selected option. For instance, in “severe climacturia + severe SUI,” AUS (T13) was selected by 83% (49/59). Similarly, high frequencies were observed in “severe climacturia + moderate SUI” and “moderate climacturia + severe SUI,” with 80% (47/59) and 81% (48/59) selecting AUS, respectively.

In scenarios involving ED requiring PPI, treatment patterns differed from those observed in SUI-dominated conditions. Among mild presentations, behavioral modifications + penile prosthesis and pelvic floor muscle training + penile prosthesis were frequently selected (42% and 44%, respectively, in “mild climacturia + ED requiring PPI”). In contrast, the Mini-Jupette procedure (T10) showed higher frequencies in more complex scenarios. Mini-Jupette was selected by 59% (35/59) in “mild climacturia + ED requiring PPI,” by 56% (33/59) in “moderate climacturia + ED requiring PPI,” and by 53% (31/59) in “severe climacturia + ED requiring PPI.”

When examining the most frequently selected treatment for each of the 24 scenarios, pelvic floor muscle training was the most common option in mild isolated presentations, male slings in moderate SUI-associated scenarios, AUS in all severe SUI scenarios, and the Mini-Jupette procedure in climacturia associated with ED requiring penile prosthesis. These values correspond directly to the detailed frequencies reported in the treatment matrix and composite figures (Table 2, Table 3, Fig. 1, Fig. 2).

## 4 Discussion

This international survey underscores how climacturia remains an underrecognized and inconsistently managed component of postprostatectomy survivorship. Despite the growing attention to disorders of ejaculation and orgasm, climacturia continues to be supported by limited evidence, fragmented definitions, and an absence of standardized outcome measures. These limitations were already emphasized in the recent Fifth International Consultation on Sexual Medicine (ICSM) review [19], which explicitly notes that climacturia is “troublesome,” common after prostate surgery, and managed with a heterogeneous mixture of behavioral, physical therapy, and surgical interventions, yet lacking validated assessment tools or robust comparative data. Our findings are consistent with, and further contextualize, this recognized gap.

Although most clinicians in our survey correctly defined climacturia and recognized that it may occur independently of SUI, routine assessment remains inconsistent. Only 59% reported evaluating climacturia in standard clinical practice, and the true rate is likely lower. Because responses were collected within a questionnaire specifically focused on climacturia, participants may have overstated their routine inquiry, introducing response bias. This is clinically relevant, as the ICSM review reports that up to 10–37% of men without SUI may experience climacturia, suggesting that the condition may remain underdetected during routine follow-up after prostatectomy or RT.

The heterogeneity observed in severity assessment mirrors the literature, which consistently notes the absence of validated climacturia-specific instruments. Experts prioritized frequency, leakage volume, and patient bother as key domains, supporting the conceptual basis of the proposed Climacturia Assessment Tool. Nevertheless, the tool should be regarded as preliminary and hypothesis-generating, and prospective studies are required to evaluate its reliability, responsiveness, and validity across different clinical contexts.

Treatment patterns across the 24 clinical scenarios revealed a pragmatic approach shaped largely by symptom severity and comorbid conditions. Conservative strategies predominated in mild or isolated cases, in line with ICSM recommendations, favoring behavioral interventions, pelvic floor muscle training, and mechanical devices. In contrast, management of moderate and severe presentations was closely aligned with established SUI or ED treatment pathways: male slings were favored in moderate SUI, while AUS implantation was consistently selected in severe cases. This pattern suggests that clinicians often address underlying incontinence or ED rather than climacturia itself when symptoms escalate, reflecting the absence of interventions specifically supported by climacturia-focused evidence. In scenarios involving ED requiring penile prosthesis, the Mini-Jupette graft [20] emerged as the preferred option. This aligns with contemporary reports describing favorable resolution rates, although long-term data remain limited, reinforcing its role as a promising but still evolving strategy.

Collectively, these findings depict a clinically intuitive management landscape that remains largely guided by expert opinion. They emphasize the need for prospective studies to clarify which interventions directly improve climacturia and to standardize outcome reporting using clinically meaningful domains.

This study has several limitations. First, the survey design is inherently limited by subjective perception, recall bias, and the hypothetical nature of clinical scenarios. Specifically, the absence of predefined patient demographics or standardized clinical scenarios represents a limitation, as participants were required to rely on their own clinical interpretation when evaluating combinations of climacturia, SUI, and ED severity, potentially introducing variability in treatment preferences. Moreover, because the survey was distributed through professional networks without tracking the total number of recipients, the response rate could not be determined, precluding formal assessment of nonresponse bias. Importantly, the findings reflect practice patterns and preferences within a specialized community rather than population-level practice or formal guideline recommendations, and responses may not fully mirror real-world behavior, particularly for rarely used interventions. Second, because participation was voluntary and drawn from a focused reconstructive and sexual medicine network, generalizability to broader urological practice is limited. Third, given the targeted focus on climacturia within the survey, contextual priming may have influenced responses, and the reported frequency of routine inquiry is likely overestimated; real-world assessment rates may be lower. Fourth, the categorization of urine leakage volume in the proposed assessment tool is based on patient-reported estimates rather than objective measurement, reflecting routine clinical practice where formal quantification during orgasm is not feasible. Although objective approaches such as condom weighing after intercourse could theoretically be considered, they remain impractical and potentially inaccurate. Finally, the proposed assessment tool is preliminary and unvalidated, and should be regarded as exploratory and hypothesis-generating rather than ready for immediate clinical implementation. Future work should include formal psychometric validation of the Climacturia Assessment Tool, including evaluation of internal consistency, test-retest reliability, construct validity, responsiveness, and correlation with established quality-of-life measures.

Interpretation of treatment patterns should also be cautious. Across scenarios, therapeutic choices appear largely driven by coexisting SUI or ED, particularly in moderate and severe presentations, suggesting that management of climacturia often defaults to established SUI or ED treatment paradigms. Although the survey included scenarios reflecting varying symptom combinations and severity, it was not designed to formally assess whether timing of surgical intervention differed according to these factors. In clinical practice, a period of conservative management is often reasonable, as functional recovery after RP, including improvements in continence and sexual function, may continue over time and potentially influence climacturia symptoms. These observations underscore the current absence of climacturia-specific evidence-based interventions and highlight an important gap in the literature.

Despite these limitations, our survey provides a comprehensive overview of current knowledge, practice patterns, and unmet needs in climacturia management. The alignment between our findings and the ICSM expert consensus supports the emerging conceptual framework and identifies opportunities for greater standardization. The proposed assessment tool and scenario-based treatment matrix should be viewed as a foundation to inform future research, including prospective validation studies and clinical trials. Ultimately, improving recognition and management of climacturia will require coordinated efforts, including rigorous validation of assessment instruments, generation of high-quality evidence for therapeutic strategies, and increased attention to climacturia as a distinct and clinically meaningful component of postprostatectomy survivorship.

  ***Author contributions*:** Malte W. Vetterlein had full access to all the data in the study and takes responsibility for the integrity of the data and the accuracy of the data analysis.

  *Study concept and design*: Chierigo, Campos-Juanatey, Ralph, Vetterlein.

*Acquisition of data*: Chierigo.

*Analysis and interpretation of data*: Chierigo.

*Drafting of the manuscript*: Chierigo, Bucca, Mantica.

*Critical revision of the manuscript for important intellectual content*: Mantica, Drocchi, Madec, Verla, Białek, Adamowicz, Cocci, Frankiewicz, Klemm, Neuville, Oszczudłowski, Redmond, Rosenbaum, Waterloos, Juanatey, Ralph, Ferro, Vetterlein.

*Statistical analysis*: Chierigo.

*Obtaining funding*: None.

*Administrative, technical, or material support*: Campos-Juanatey, Ralph, Ferro, Vetterlein.

*Other* (specify): None.

  ***Financial disclosures:*** Malte W. Vetterlein certifies that all conflicts of interest, including specific financial interests and relationships and affiliations relevant to the subject matter or materials discussed in the manuscript (eg, employment/affiliation, grants or funding, consultancies, honoraria, stock ownership or options, expert testimony, royalties, or patents filed, received, or pending), are the following: None.

  ***Funding/Support and role of the sponsor*:** None.

  ***Acknowledgments:*** Trauma and Reconstructive Urology Working Party of the European Association of Urology Young Academic Urologists collaborators: Behzad Abbasi, Leonidas Karapanos, Mattia Lo Re, Jordán Scherñuk, Juan Diego Tinajero. European Association of Urology Section of Genitourinary Reconstructive Surgeons collaborators: Mark Joseph Abalajon, Hussain M. Alnajjar, Mario Álvarez-Maestro, Carlo Bettocchi, Ozan Bozkurt, Fabiana Cancrini, Luisana Castillo Carvajal, Gökhan Çeker, Bülent Çetinel, Mehmet Gokhan Culha, Sabrina De Cillis, Muslim Dogan Deger, Rados Djinovic, Marco Falcone, Lufcas Freton, Paolo Geretto, Jean-Pierre Graziana, Cyrille Guillot-Tantay, Francois Hervé, Charalampos Konstantinidis, Celeste Manfredi, Sachin Malde, José Medina-Polo, Lionel A. Micol, Andrey Morozov, Jan Novák, Yanira Ortega, Marie-Aimée Perrouin-Verbe, Arthur Peyrottes, Mikołaj Przydacz, Giorgio I. Russo, Koenraad van Renterghem, Stephane de Vergie, Patrizio Vicini, Sam Ward, Faysal Yafi

## Figures and Tables

**Supplementary Figure 3:**
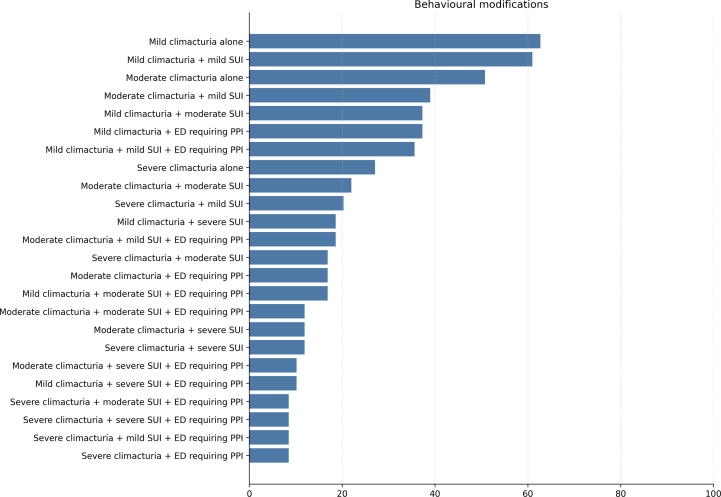
Plot of expert recommendation on the use of behavioral modifications according to combinations of different severity levels (mild, moderate, severe) of climacturia, stress urinary incontinence (SUI), and erectile dysfunction (ED)

**Supplementary Figure 4:**
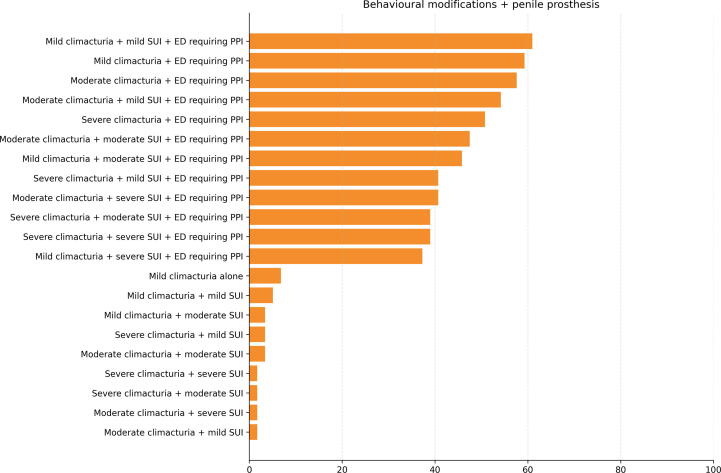
Plot of expert recommendation on the use of behavioral modifications + penile prosthesis implantation according to combinations of different severity levels (mild, moderate, severe) of climacturia, stress urinary incontinence (SUI), and erectile dysfunction (ED)

**Supplementary Figure 5:**
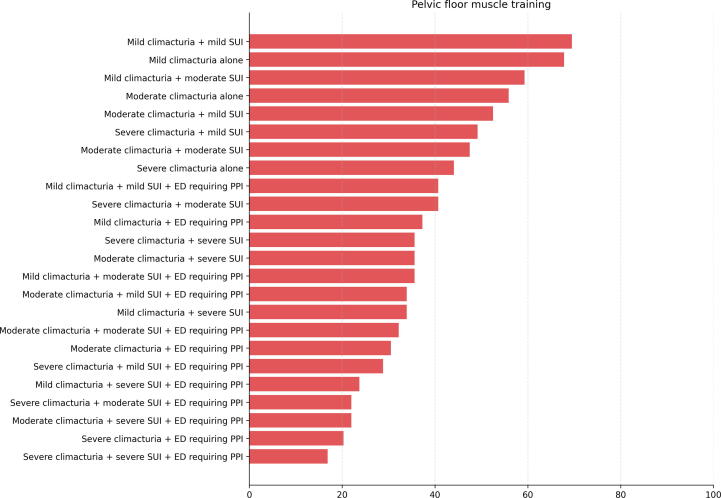
Plot of expert recommendation on the use of pelvic floor muscle training according to combinations of different severity levels (mild, moderate, severe) of climacturia, stress urinary incontinence (SUI), and erectile dysfunction (ED)

**Supplementary Figure 6:**
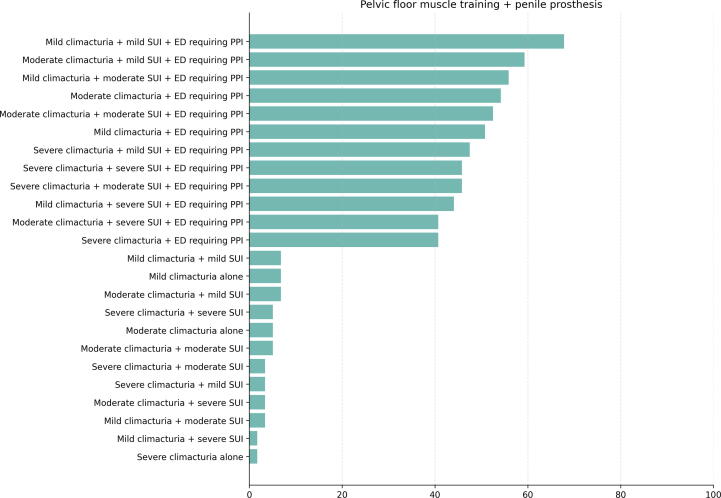
Plot of expert recommendation on the use of pelvic floor muscle training + penile prosthesis implantation according to combinations of different severity levels (mild, moderate, severe) of climacturia, stress urinary incontinence (SUI), and erectile dysfunction (ED)

**Supplementary Figure 7:**
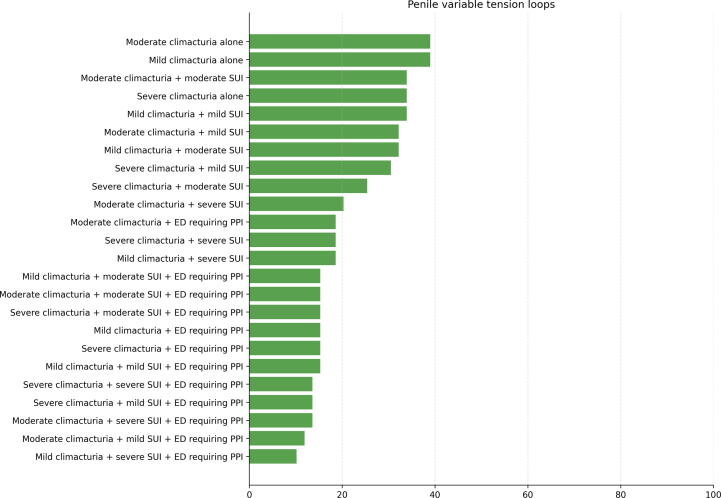
Plot of expert recommendation on the use of variable tension loops according to combinations of different severity levels (mild, moderate, severe) of climacturia, stress urinary incontinence (SUI), and erectile dysfunction (ED)

**Supplementary Figure 8:**
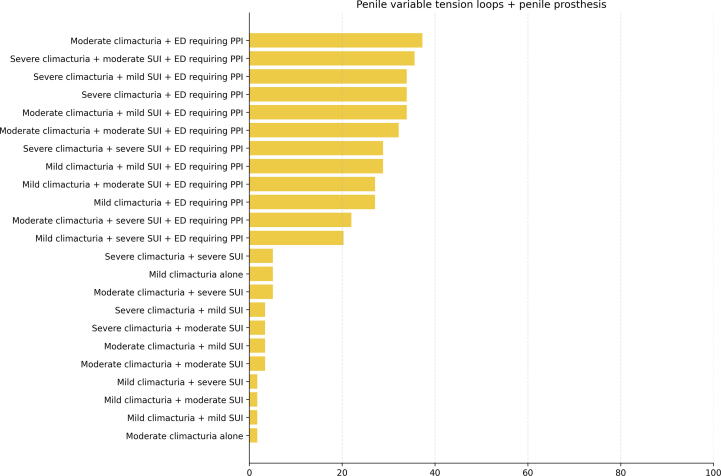
Plot of expert recommendation on the use of variable tension loops + penile prosthesis implantation according to combinations of different severity levels (mild, moderate, severe) of climacturia, stress urinary incontinence (SUI), and erectile dysfunction (ED)

**Supplementary Figure 9:**
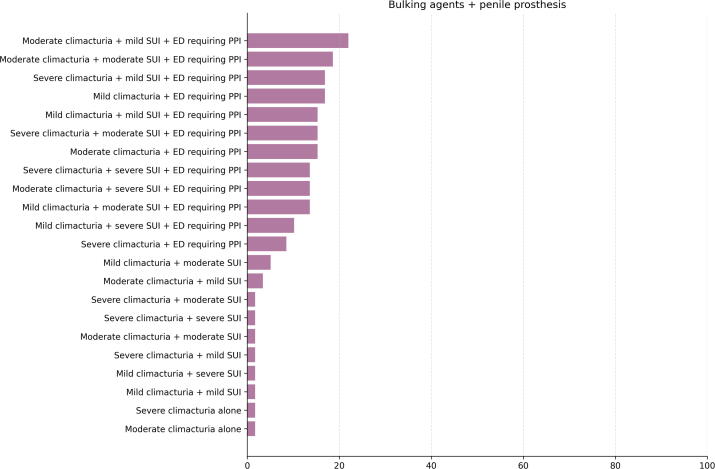
Plot of expert recommendation on the use of bulking agents + penile prosthesis implantation according to combinations of different severity levels (mild, moderate, severe) of climacturia, stress urinary incontinence (SUI), and erectile dysfunction (ED)

**Supplementary Figure 10:**
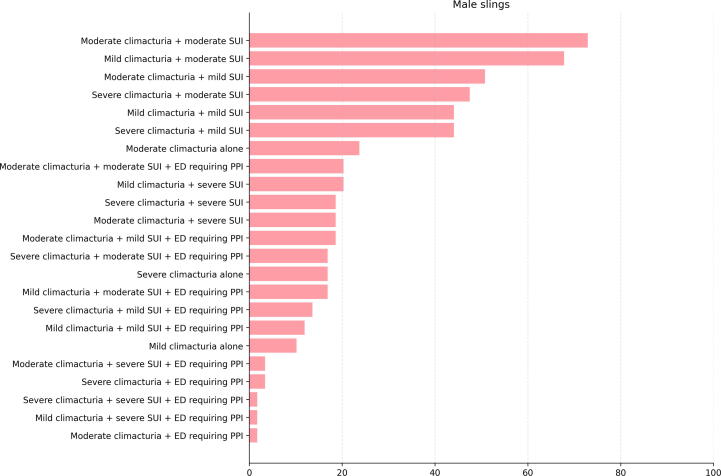
Plot of expert recommendation on the use of male slings according to combinations of different severity levels (mild, moderate, severe) of climacturia, stress urinary incontinence (SUI), and erectile dysfunction (ED)

**Supplementary Figure 11:**
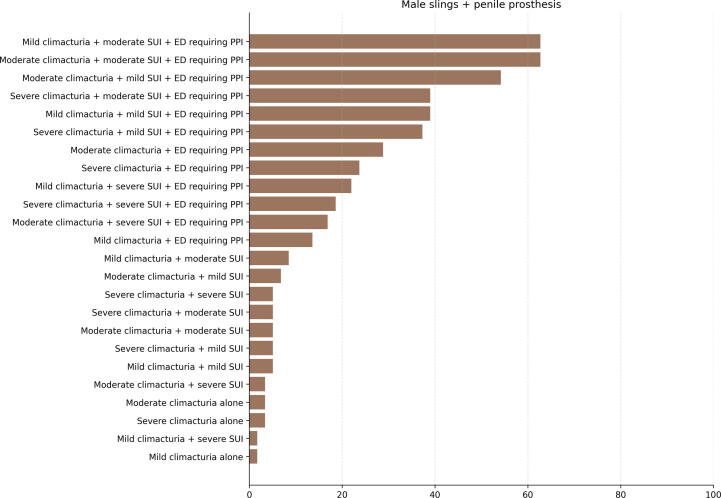
Plot of expert recommendation on the use of male slings + penile prosthesis implantation according to combinations of different severity levels (mild, moderate, severe) of climacturia, stress urinary incontinence (SUI), and erectile dysfunction (ED)

**Supplementary Figure 12:**
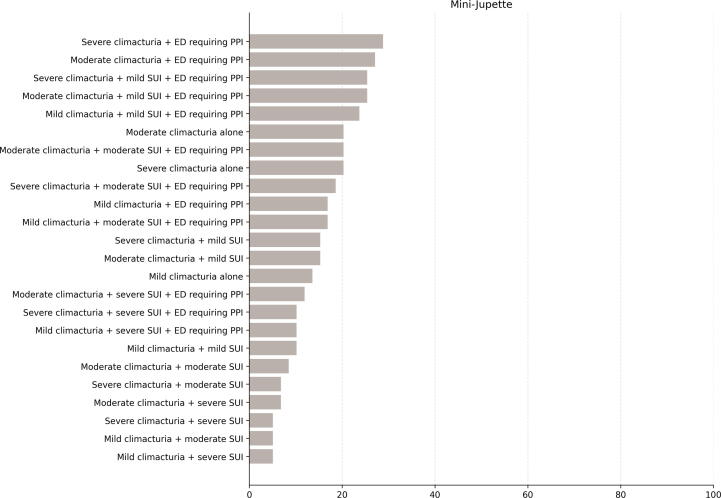
Plot of expert recommendation on the use of Mini-Jupette according to combinations of different severity levels (mild, moderate, severe) of climacturia, stress urinary incontinence (SUI), and erectile dysfunction (ED)

**Supplementary Figure 13:**
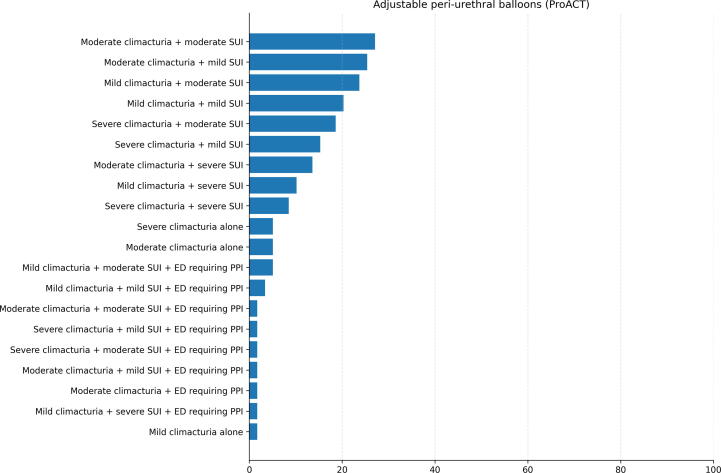
Plot of expert recommendation on the use of adjustable peri-urethral balloons according to combinations of different severity levels (mild, moderate, severe) of climacturia, stress urinary incontinence (SUI), and erectile dysfunction (ED)

**Supplementary Figure 14:**
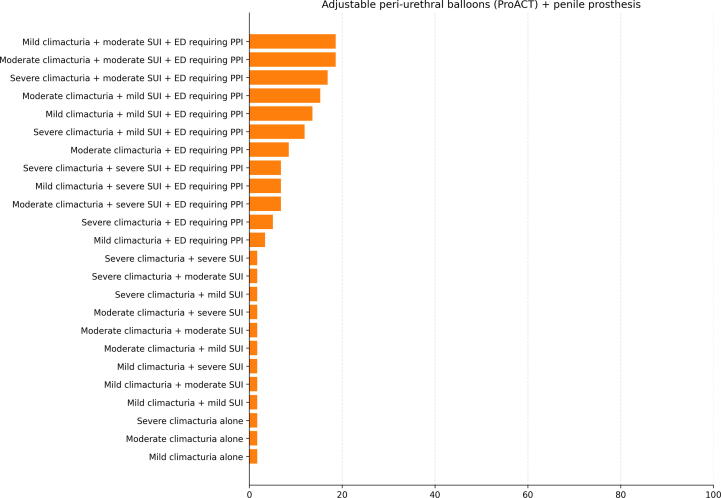
Plot of expert recommendation on the use of adjustable peri-urethral balloons + penile prosthesis implantation according to combinations of different severity levels (mild, moderate, severe) of climacturia, stress urinary incontinence (SUI), and erectile dysfunction (ED)

**Supplementary Figure 15:**
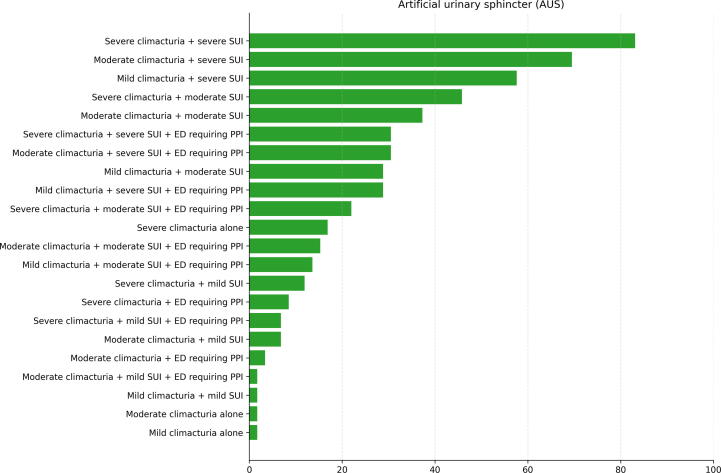
Plot of expert recommendation on the use of artificial urinary sphincter according to combinations of different severity levels (mild, moderate, severe) of climacturia, stress urinary incontinence (SUI), and erectile dysfunction (ED)

**Supplementary Figure 16:**
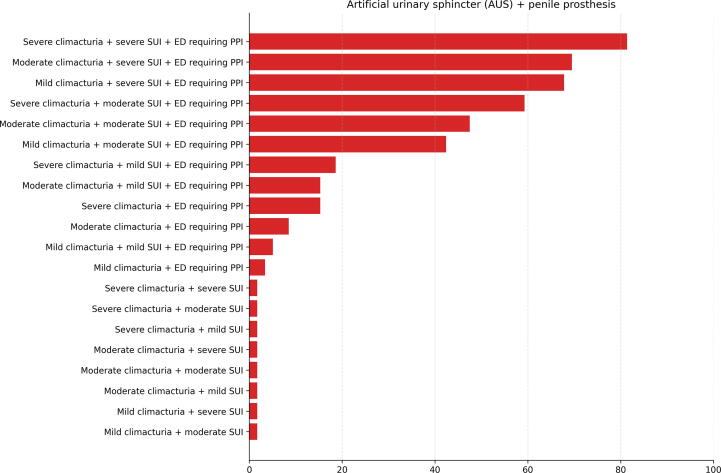
Plot of expert recommendation on the use of artificial urinary sphincters + penile prosthesis implantation according to combinations of different severity levels (mild, moderate, severe) of climacturia, stress urinary incontinence (SUI), and erectile dysfunction (ED)

## Appendix A Supplementary material

Supplementary Data 1 Climacturia Survey

Supplementary Data 2 Climacturia Assessment Tool
