# Supplementary material for: Contemporary Practices in the Assessment and Management of Climacturia: Results from an International Survey
Source: Eur Urol Open Sci. 2026 Jun 17;90:20–31. doi: 10.1016/j.euros.2026.05.004 (PMC13310649; doi:10.1016/j.euros.2026.05.004)
Supplement: Supplementary Data 2 — Climacturia Assessment Tool [file mmc2.docx]

**Climacturia Assessment Tool**

**1. Urine Loss Volume (0-3 points)**

- **0 points**: None
- **1 point**: Small drops
- **2 points**: <30 mL
- **3 points**: ≥30 mL

**2. Frequency of Urine Loss During Climax (0-3 points)**

- **0 points**: None
- **1 point**: 25% of the time
- **2 points**: 50% of the time
- **3 points**: 75% or always

**3. Patient Bother (0-6 points)**

- **0 points**: Not bothersome at all
- **2 points**: Slightly bothersome
- **4 points**: Moderately bothersome
- **6 points**: Extremely bothersome

**Total Score Calculation:**

- Total score = (Urine Loss Volume) + (Frequency) + (Patient Bother)

**Severity Categories:**

- **0 points**: No climacturia
- **1-4 points**: Mild climacturia
- **5-8 points**: Moderate climacturia
- **9-12 points**: Severe climacturia
