# Supplementary material for: Contemporary Practices in the Assessment and Management of Climacturia: Results from an International Survey
Source: Eur Urol Open Sci. 2026 Jun 17;90:20–31. doi: 10.1016/j.euros.2026.05.004 (PMC13310649; doi:10.1016/j.euros.2026.05.004)
Supplement: Supplementary Data 1 — Climacturia Survey [file mmc1.docx]

YAU / ESGURS Survey on Climacturia

Dear Prof. / Colleague,

Climacturia is a neglected post-prostatectomy side effect. The literature about climacturia is scant, with only 68 publications on PubMed.

In absence of contemporary literature on its prevalence and only 13 studies on possible treatments of climacturia, with no comparative studies available, it is of utmost importance that scientifi c societies, such as the EAU Section of Genito-Urinary Reconstructive Surgery and the YAU Trauma and reconstructive urology working party, join forces to fi ll the existing knowledge gap.

In this context, we aim to introduce a multistep research endeavor to reach consensus on the assessment of climacturia and its treatment according to symptom severity.

The first step of this research would be understanding how experts like you manage climacturia by replying to the following survey.

Following the feedback given by the participants, the future step could be:

1. A climacturia multicentric database to study its prevalence, its association with other post-prostatectomy side effects, and its treatment.

2. A Delphi consensus on the treatment strategy according to symptom severity

All participants who will complete all the required rounds will be listed as Pubmed Collaborators in the upcoming publication(s).

Please don't share it! At the end of the survey you will be given the chance to advice some colleagues expert in climacturia treatment.

We would like to thank you for your time and effort.

Kind regards,

F. Chierigo, G. Mantica, W. Verla, F. Campos Juanatey, J. Adamowicz, A. Cocci, M. Frankiewicz,C.M. Rosenbaum, M. Waterloos, E. Redmond, FX. Madec, M. Vetterlein EAU - YAU Trauma and reconstructive urology working party

General information

1.	Email

2.	What is your age?

3.	What is your role?

- Junior doctor

- Resident

- Consultant

- Other (specify)

4.	Working setting

- Teaching hospital

- Non-teaching hospital

5.	Do you personally perform surgery for male urinary incontinence?

- Yes

- No

6.	Do you personally perform penile implant surgery?

- Yes

- No

7.	Does your hospital have a dedicated clinic for post-prostatectomy patients?

- Yes

- No

8.	In post-prostatectomy patients, do you routinely ask for presence of urinary incontinence?

- Yes

- No

9.	How do you grade urinary incontinence?

- 24-hour pad test

- ICIQ-UI-SF

- Pads per day

- MSIGS

- Other (specify)

10.	In post-prostatectomy patients, do you routinely ask for presence of erectile dysfunction?

- Yes

- No

11.	How do you grade post-prostatectomy erectile function?

- IIEF / IIEF-5

- Ability to achieve sufficient erection for penetrative intercourse

- Erection Hardness Score

- Other (specify)

12.	In post-prostatectomy patients, do you routinely ask for presence of orgasmic dysfunction?

- Yes

- No

Climacturia - definition, classification and prevalence

13. Do you know what climacturia is?

- Yes

- No

14.	Could you provide a definition of climacturia? (open reply)

15.	Do you believe that climacturia can present independently of stress urinary	incontinence?

- Yes

- No

16.	How do you usually assess the severity of climacturia? (open reply)

17.	On a scale from 1 to 9 (range from 1, “very bad”, to 9, “very well”) how good do	* you think the following grading of climacturia severity (based on volume of urine) works?

-	No climacturia = Dry

-	Mild climacturia = Few drops

-	Moderate climacturia = <30mL

-	Severe climacturia = >=30mL

18.	In case you do not agree with the previous scale, how would you modify the previous definitions? (open reply)

19.	On a scale from 1 to 9 (range from 1, “very bad”, to 9, “very well”) how much do you think the following grading of climacturia severity (based on frequency of climacturia) works?

-	None = never

-	Mild = around 25% of the times

-	Moderate = around 50% of the times

-	Severe = around 75% of the times

-	Very severe = always

20.	In case you do not agree with the previous scale, how would you modify the previous definitions? (open reply)

21.	On a scale from 1 to 9 (range from 1, “very bad”, to 9, “very well”) how much do you think the following OVERALL grading of climacturia works?

Urine loss

0	points -None

1	point - small drops

2	points - <30mL

3	points - >=30mL

Frequency

0	points - None

1	point - 25% of times

2	points - 50% of times

3	points - 75% of times

4	points - always

Total = urine loss + frequency

0 no climacturia

1-3 mild climacturia

4-5 moderate climacturia

6-7 severe climacturia

22.	In case you do not agree with the previous scale, how would you modify the previous definitions? (open reply)

23.	In your experience, what is the prevalence of climacturia alone at 3 months after RP? (0-100%)

24.	In your experience, what is the prevalence of climacturia + stress UI at 3 months after RP? (0-100%)

25.	In your experience, what is the prevalence of climacturia + ED at 3 months after RP? (0-100%)

26.	In your experience, what is the prevalence of climacturia + stress UI + ED at 3 months after RP? (0-100%)

27.	In your experience, what is the prevalence of climacturia alone at 12 months after RP? (0-100%)

28.	In your experience, what is the prevalence of climacturia + stress UI at 12	months after RP? (0-100%)

29.	In your experience, what is the prevalence of climacturia + ED at 12 months after RP? (0-100%)

30.	In your experience, what is the prevalence of climacturia + stress UI + ED at 12 months after RP? (0-100%)

Climacturia - Treatment options

31.	Do you perform surgery for climacturia?

- Yes

- No

32. What do you feel is the correct timing to perform surgery for climacturia?

33. For which patient would you consider behavioural modifications (condoms, emptying the bladder before intercourse, etc.)? (the options are the same for Q33->46, more than one option available)

- Mild climacturia alone

- Moderate climacturia alone

- Severe climacturia alone

- Mild climacturia + mild SUI

- Mild climacturia + moderate SUI

- Mild climacturia + severe SUI

- Moderate climacturia + mild SUI

- Moderate climacturia + moderate SUI

- Moderate climacturia + severe SUI

- Severe climacturia + mild SUI

- Severe climacturia + moderate SUI

- Severe Climacturia + severe SUI

- Mild climacturia + ED requiring PPI

- Moderate climacturia + ED requiring PPI

- Severe climacturia + ED requiring PPI

- Mild climacturia + mild SUI + ED requiring PPI

- Mild climacturia + moderate SUI + ED requiring PPI

- Mild climacturia + severe SUI + ED requiring PPI

- Moderate climacturia + mild SUI + ED requiring PPI

- Moderate climacturia + moderate SUI + ED requiring PPI

- Moderate climacturia + severe SUI + ED requiring PPI

- Severe climacturia + mild SUI + ED requiring PPI

- Severe climacturia + moderate SUI + ED requiring PPI

- Severe climacturia + severe SUI + ED requiring PPI

- None

- Other (specify)

34. For which patient would you consider behavioural modifications (condoms, emptying the bladder before intercourse, etc.) + penile prosthesis implantation?

35. For which patient would you consider pelvic floor muscle training?

36. For which patient would you consider pelvic floor muscle training + penile prosthesis implantation?

37. For which patient would you consider   penile variable tension loops/soft silicone tension loops?

38. For which patient would you consider   penile variable tension loops/soft silicone tension loops + penile prosthesis implantation?

39. For which patient would you consider bulking agents + penile prosthesis implantation?

40. For which patient would you consider   male slings?

41. For which patient would you consider   male slings + penile prosthesis implantation?

42. For which patient would you consider Mini-Jupette?

43. For which patient would you consider adjustable peri-urethral baloons (i.e. ProACT)?

44. For which patient would you consider adjustable peri-urethral baloons (i.e. ProACT) + penile prosthesis implantation?

45. For which patient would you consider   artificial urinary sphincter (AUS)?

46. For which patient would you consider   artificial urinary sphincter (AUS) + penile prosthesis implantation?
